# Associations of thyroid feedback quantile-based index with diabetes in euthyroid adults in the United States and China

**DOI:** 10.1080/07853890.2024.2318418

**Published:** 2024-02-21

**Authors:** Heng Wan, Genfeng Yu, Yajun He, Siyang Liu, Xingying Chen, Yuqi Jiang, Hualin Duan, Xu Lin, Lan Liu, Jie Shen

**Affiliations:** a Department of Endocrinology and Metabolism, Shunde Hospital, Southern Medical University (The First People’s Hospital of Shunde, Foshan), Foshan, Guangdong, China; b School of Nursing, Southern Medical University, Guangzhou, Guangdong, China

**Keywords:** Impaired sensitivity to thyroid hormones, diabetes, TFQI

## Abstract

**Background:**

We aimed to investigate the associations between thyroid hormone sensitivity indices and diabetes in euthyroid adults in the United States and China.

**Methods:**

2296 euthyroid adults from the NHANES in the United States and 8319 euthyroid adults from the SPEED-Shunde in China were involved. The thyroid sensitivity indices, namely TFQIFT4 and TFQIFT3, were calculated. Multivariable logistic regression, restricted cubic spline analysis, and general ordinal logit regression were utilized.

**Results:**

In the NHANES, compared with participants in quartile 1st (Q1), those in Q4 of TFQI_FT3_ (OR 2.12, 95% CI (1.18, 3.81)) and those in Q3 of TFQI_FT4_ (OR 2.31, 95% CI (1.18, 4.53)) (both *P*_for trend_ < 0.05) were associated with a greater prevalence of diabetes. In the SPEED-Shunde, compared with participants in Q1, those in Q4 of TFQI_FT3_ had a greater prevalence of diabetes (OR 1.36, 95% CI (1.11, 1.66) (*P*_for trend_ < 0.05), while no significant associations between TFQI_FT4_ and diabetes were found.

**Conclusions:**

TFQI_FT3_ was associated with a higher prevalence of diabetes both in the United States and China. However, TFQI_FT4_ was only associated with a higher prevalence of diabetes in the United States, not in China. Further prospective cohort studies are necessary to validate these findings.

## Introduction

Diabetes has become a critical global health problem due to its high prevalence and associated disability and mortality. The prevalence of diabetes in China, the USA, and worldwide was 11.7%, 11.6% and 6.1% [1,2], respectively, resulting in global health expenditure of US$966 billion globally, forecast to reach more than $1054 billion by 2045 [2]. Thyroid hormones (THs) and thyroid-stimulating hormone (TSH) have been widely recognized as significant endocrine regulators of glucose metabolism [3,4]. The presence of both hypothyroidism and hyperthyroidism has been proposed to be linked to the development of diabetes [5,6]. Significant associations were also found between subclinical hypothyroidism or hyperthyroidism with metabolic health [7,8]. However, the metabolic mechanisms previously elucidated in clinical or subclinical thyroid diseases may not comprehensively explain the observed associations within the normothyroid range.

Recent findings indicate a prevalent occurrence of relatively elevated levels of both TSH and THs in euthyroid adults, suggesting impaired central sensitivity to THs [9,10], although there is a physiologically inverse correlation between FT4 and FT3 with TSH due to a negative feedback loop [11]. Laclaustra et al. have proposed a novel thyroid feedback quantile-based index (TFQI) that explicitly targets deviations from normal thyroid hormone values (rather than extreme ranges) to identify impaired central sensitivity to THs within the general population [12]. However, the relationships between TFQI and diabetes or abnormal glucose metabolism displayed inconsistency across various populations of different race/ethnicity. A positive correlation between TFQI and the prevalence of diabetes was observed in a sample size of 5,129 individuals, with Asians constituting less than 4.5% of the total population [12]. In contrast, a comprehensive multicentre retrospective study involving 30,244 Chinese participants revealed a significant inverse association between TFQI and the prevalence of elevated blood glucose levels, encompassing both prediabetes and diabetes [13]. Additionally, an earlier study with a sample size of 4378 individuals also indicated that higher TFQI was associated with a lower risk of prediabetes [14]. Indeed, the influence of race/ethnicity on the parameters of TSH and thyroid hormones has been firmly established [15–17]. Consequently, it is imperative to assess the stability of the correlation between TFQI and diabetes across diverse racial populations, employing identical exclusion criteria and outcome definitions.

TFQI, commonly called impaired central sensitivity to FT4 (TFQI_FT4_), is determined by assessing FT4 and TSH levels [12]. Since the inhibitory effect of FT3 on TSH has been established [18], we previously substituted FT4 with FT3 in the TFQI_FT4_ formulas and revealed that TFQI_FT3_, rather than TFQI_FT4_, exhibited a positive association with the prevalence of metabolic dysfunction-associated fatty liver disease (MAFLD) in euthyroid Chinese adults [19]. However, the studies on the associations between TFQI_FT3_ and diabetes were limited, especially in diverse racial populations. Furthermore, the impact of peripheral sensitivity to thyroid hormones, expressed by the FT3/FT4 ratio, has gained significant attention concerning diabetes [4,20,21].

Taken together, the objective of this study was to investigate the associations of thyroid hormone sensitivity indices, specifically TFQI_FT4_, TFQI_FT3_ and FT3/FT4 ratio, with diabetes in euthyroid adults in the United States and China through two cross-sectional community-based surveys.

## Method

### Study design and population enrolment

**Figure 1.**
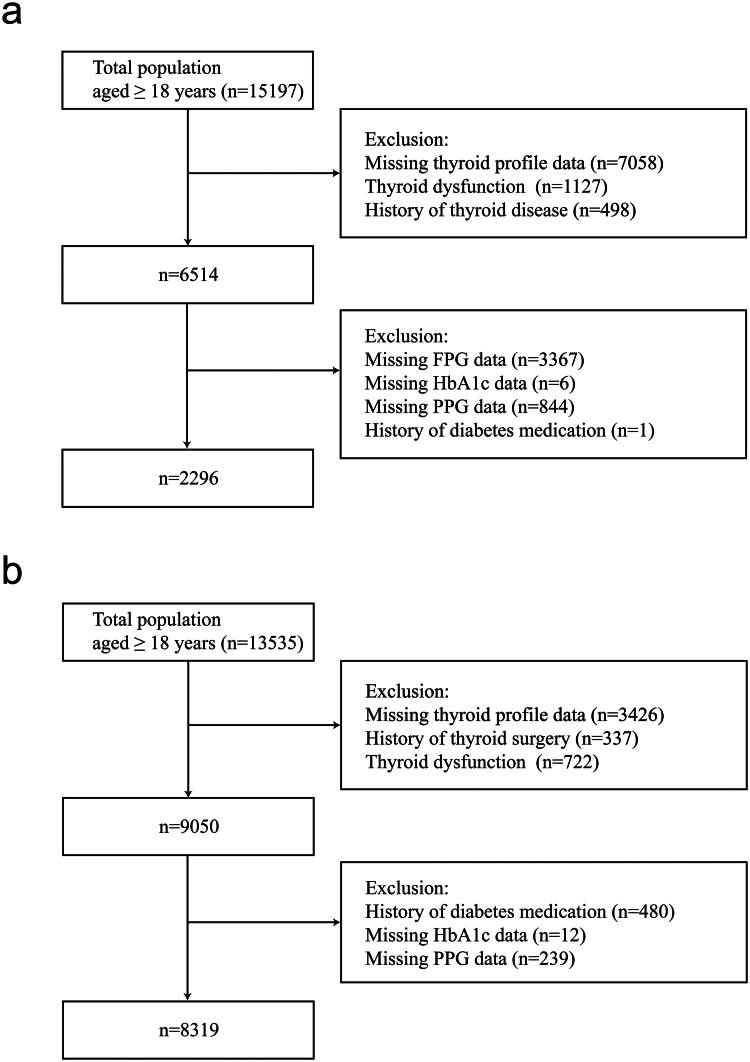
Flowchart of the study participants. (a) The NHANES. (b) The SPEED-Shunde.

Our current cross-sectional study used two databases from the USA and China. Euthyroid individuals from the National Health and Nutrition Examination Survey (NHANES) during the period from 2007 to 2012 were enrolled. The NHANES is a complex, multi-stage probability sampling survey that contains abundant data on demographics, physical examinations, laboratory samples, lifestyle and self-report health status, conducted by the National Center for Health Statistics, part of the Centers for Disease Control and Prevention [22,23]. NHANES data files are released publicly every two years. We combined three NHANES cycles (i.e. 2007–2008, 2009–2010 and 2011-2012) following the NHANES guidelines. In the present study, we analysed NHANES data, including adults aged ≥18 years (*n* = 15197). We excluded participants whose thyroid function data were not available (*n* = 7058), those who with thyroid dysfunction (*n* = 1127) or a history of thyroid disease (*n* = 498), whose fasting plasma glucose (FPG) data were missing (*n* = 3367), whose HbA1c data were missing (*n* = 6), whose 2-h postprandial plasma glucose (PPG) data were missing (*n* = 844) and those who with the history of diabetes medication (*n* = 1). Finally, 2296 participants were included in this analysis (Figure 1(a)).

The Prevalence of Metabolic Diseases and Risk Factors in Shunde (SPEED-Shunde) is a community-based study on the prevalence of metabolic diseases and risk factors in Shunde District, Foshan City, Guangdong Province, South China. It was conducted between November 2021 and September 2022 in persons ≥18 years old and who had lived in Shunde for at least six months from ten study sites using a stratified cluster sampling method. The registration number is ChiCTR2100054130 (www.chictr.org). The specific method is as described before [19]. 13535 subjects aged 18 years or older were invited to participate during the recruiting phase. We excluded participants with the missing values of thyroid profiles (*n* = 3426), with a history of thyroid surgery (*n* = 337), with thyroid dysfunction (*n* = 722), with a history of diabetes medication (*n* = 480), with the missing value of HbA1c data (*n* = 12) and with the missing value of PPG data (*n* = 239). Finally, 8319 subjects were eligible for inclusion in the present study (Figure 1(b)).

### Demographic, behavioural and anthropometric assessment

Participants self-reported the following covariates: age, sex, race/ethnicity, medication information, education level and health behaviours, including smoking and alcohol consumption. The body mass index (BMI) of the subjects was calculated as weight (kilograms) divided by height (meters) squared (kg/m^2^).

### Laboratory tests

The NHANES study provided detailed information on laboratory methods used to measure various biomarkers, including FPG, glycated haemoglobin (HbA1c), PPG, FT3, FT4, TSH, thyroid peroxidase antibody (TPOAb), thyroglobulin antibody (TgAb), creatinine, total cholesterol (TC), triglyceride (TG), high-density lipoprotein cholesterol (HDL-C), low-density lipoprotein cholesterol (LDL-C), were reported as previously documented [24]. Serum TSH was measured by microparticle enzyme immunoassay, serum FT4 by two-step enzyme ­immunoassay and serum FT3 by competitive binding immunoassay [25,26].

In the SPEED-Shunde study, blood samples were collected between 7:00 am and 10:00 am from participants who had fasted for 10 h. The levels of FT3, FT4, TSH, TPOAb and TgAb in the serum were measured using the Microparticle Enzyme Immunoassay (MEIA) technique on the UniCel Dxi 800 Access instrument from Beckman Coulter, USA. FPG, PPG, creatinine, TC, TG, HDL-C and LDL-C were analysed using the automatic biochemical analyser AU5831 from Beckman Coulter, USA. HbA1c levels were determined using high-performance liquid chromatography (HLC-723G8, TOSOH, Japan), while fasting insulin was assessed by an automatic chemiluminescence immunoassay analyser, CL-6000i (Mindray, China). All samples were shipped under cold chain management to the central laboratory in Shunde Hospital, centrifuged and frozen at −20 °C within 2 h.

### Definition of serum thyroid function

The normal reference ranges for TSH, FT4 and FT3 are 0.39–4.60 mIU/L, 0.6–1.6 ng/dL (7.8–20.8 pmol/L) and 2.5–3.9 pg/mL (3.85–6.006 pmol/L), respectively [26] in the NHANES and 0.56-5.91 mIU/L, 7.98-16.02 pmol/L and 3.53-7.37 pmol/L, respectively in the SPEED-Shunde study [19]. Euthyroid was defined as TSH, FT4 and FT3 were all within the normal range. TFQI_FT4_ was obtained by the algorithm TFQI_FT4_ = cumulative distribution function (cdfFT4) − (1 − cdfTSH) and TFQI_FT3_ was obtained by TFQI_FT3_ = cumulative distribution function (cdfFT3) − (1 − cdfTSH). The value of TFQI ranged from −1 to 1. Negative values indicate greater sensitivity to thyroid hormones, and positive values indicate less sensitivity.

### Definitions of diabetes and other covariates

Baseline hypertension was defined as systolic blood pressure (SBP) ≥140 mmHg, diastolic blood pressure (DBP) ≥90 mmHg, or self-reported use of antihypertensive medication. The definition of diabetes was FPG level ≥7.0 mmol/L, PPG ≥11.1 mmol/L, HbA1c ≥6.5% and/or having a self-reported diagnosis of diabetes as in the previous study [1]. Glucose status was defined as a three-level categorical variable as diabetes, prediabetes (i.e. FPG levels 5.6 to 6.9 mmol/L, or 2-h PPG levels 7.8 to 11.0 mmol/L or HbA1c 5.7% to 6.4%) and normoglycemia (i.e. FPG levels <5.6 mmol/L, 2-h PPG levels <7.8 mmol/L and HbA1c <5.7%) in the final included participants. Thyroiditis was defined as TPOAb or TGAb positive (TPOAb > 9.0 IU/mL or TGAb > 115.0 IU/mL in the NHANES and TPOAb > 9.0 IU/mL or TGAb > 4.9 IU/mL in the SPEED-Shunde) according to their reference ranges [27]. Dyslipidemia was identified in individuals whose TC ≥ 6.22 mmol/L, TG ≥ 2.26 mmol/L, LDL ≥ 4.14 mmol/L, or HDL < 1.04 mmol/L, or self-reported diagnosis of dyslipidemia as the previous study [28,29]. Alcohol consumption was reported as standard drinks and converted to grams by multiplying by 14. Abusive drink was defined as >30 g/day for men and >20 g/day for women [30]. Smoking status was classified as never (smoked <100 cigarettes in their lifetime), former (smoked >100 cigarettes in their lifetime but currently did not smoke at all) and current smokers (smoked >100 cigarettes in their lifetime and currently smoked some days or every day) [31]. Education was categorized into three levels: less than high school, completed high school and beyond high school [32]. The eGFR was computed through the Chronic Kidney Disease Epidemiology Collaboration (CKD-EPI) equation and estimated ­glomerular filtration rate (eGFR) = 141 × min (SCr/*κ*, 1)*^α^* × max (SCr/*κ*, 1)^−1.209^ × 0.993^Age^ × 1.018 if female × 1.159 if black [33]. HOMA-IR was assessed by the formula: HOMA-IR = FBG (mmol/L) x fasting insulin (μU/mL)/22.5 [34].

### Statistical analysis

The baseline characteristics of study participants were summarized as mean (standard deviation) or median (interquartile range) depending on the variable distribution for continuous variables, and categorical variables were presented as proportion (%).

In the SPEED-Shunde study, multivariable logistic regression was used to identify the associations of the thyroid sensitivity indices with diabetes. The full model was adjusted for sex, age, BMI, eGFR, hypertension, thyroiditis, dyslipidemia, abused drink, education, smoking status and HOMA-IR. In the NHANES study, the associations of the thyroid sensitivity indices with diabetes were investigated using the survey-weighted generalized linear model, further adjusting for race/ethnicity. No collinearity among the confounders was found (variance inflation factor < 5) [35]. A restricted cubic spline (RCS) analysis was conducted using a three-knot restricted cubic spline function (with knots at the 10th, 50th and 90th percentiles) to further validate the potential non-linear relationships of the thyroid sensitivity indices with diabetes using the fully adjusted model. Ordinal logistic regression was not suitable for the current study as detailed specification tests revealed that some variables violated the parallel regression assumption (*p* < 0.05) [36]. Therefore, a general ordinal logit regression was constructed to assess the associations of thyroid sensitivity indices with the normal/prediabetes/diabetes category using the fully adjusted model. Finally, stratified analyses by sex (men, women), age groups (<60, ≥60 years), BMI (in the NHANES study: <25, 30 > BMI ≥ 25, ≥30 kg/m^2^; in the SPEED-Shunde: <24, 28 > BMI ≥ 24, ≥28 kg/m^2^) were also performed. Interactions between thyroid sensitivity indices and subgroups on diabetes events were also tested in the models. *P* values below 0.05 were considered statistically significant. The missing values were handled using multiple imputations by mice R-package. Appropriate sampling weights were used in the NHANES (except the RCS analysis), as suggested by the NCHS. An unweighted RCS analysis was conducted, considering the R package for the weighted RCS analysis was unavailable. All statistical analyses were conducted using R (version 4.2.1).

## Results

### General characteristics of the participants

The baseline characteristics of participants from the NHANES and the SPEED-Shunde are presented in Table 1. Among the 2296 participants from the NHANES, the weighted prevalence of participants with diabetes, men, non-Hispanic whites, and non-smokers were 6.62%, 51.44%, 68.78% and 55.20%, respectively. Mean FPG, HbA1c, PPG, FT3 and FT4 in the NHANES were 5.56 mmol/L, 5.41%, 6.37 mmol/L, 4.97 pmol/L and 10.23 pmol/L, respectively, and median TSH was 1.67 mIU/L.

**Table 1.** General characteristics of participants in the study.

	NHANES	SPEED-Shunde
N	2296	8319
Age, year	44.56 (16.14)	46.48 (12.11)
Men, %	51.44	38.19
Race/ethnicity		
Mexican American	9.16	–
Other Hispanic	5.52	–
Non-Hispanic White	68.78	–
Non-Hispanic Black	9.81	–
Others	6.73	–
Education, %		
Less than high school	29.68	39.07
High school	31.20	24.10
Beyond high school	39.12	36.83
Abusive drink, %	0.19	2.95
Smoking, %		
Non-smokers	55.20	85.88
Former smokers	23.54	2.97
Current smokers	21.26	11.16
Diabetes, %	6.62	12.30
BMI, kg/m^2^	27.99 (5.97)	23.66 (3.44)
SBP, mmHg	120.34 (16.12)	123.43 (17.07)
DBP, mmHg	70.01 (11.84)	82.14 (17.91)
FPG, mmol/L	5.56 (0.78)	4.79 (1.01)
HbA1c, %	5.41 (0.48)	5.68 (0.62)
PPG, mmol/L	6.37 (2.53)	7.77 (2.90)
HOMA-IR	2.29 (1.46, 3.69)	3.92 (7.83)
FT3, pmol/L	4.97 (0.47)	5.35 (0.58)
FT4, pmol/L	10.23 (1.56)	11.26 (1.42)
TSH, mIU/L	1.67 (1.17, 2.36)	1.74 (1.24,2.43)
FT3/FT4 ratio	0.50 (0.08)	0.48 (0.07)
TFQI_FT3_	−0.02 (0.40)	−0.04 (0.39)
TFQI_FT4_	−0.04 (0.39)	−0.04 (0.38)
TPOAb, IU/ml	0.60 (0.30, 1.50)	1.00 (0.50;3.23)
TgAb, IU/ml	0.60 (0.60, 0.60)	0.10 (0.07;0.64)
eGFR, mL/min/m^2^	98.35 (19.74)	96.26 (15.76)
TG, mmol/L	1.19 (0.85, 1.71)	1.21 (0.86,1.80)
TC, mmol/L	5.07 (1.03)	5.38 (1.58)
HDL-C, mmol/L	1.39 (0.39)	1.45 (0.33)
LDL-C, mmol/L	3.03 (0.89)	3.01 (0.74)

*Notes:* The baseline characteristics of participants were summarized as mean (standard deviation) or median (interquartile range) for continuous variables and proportion (%) for categorical variables according to with or without diabetes. Appropriate sampling weights were used when calculating the data in the NHANES. BMI: body mass index; SBP: systolic blood pressure; DBP: diastolic blood pressure; FPG: fasting plasma glucose; HbA1c: Glycated haemoglobin; PPG: postprandial glucose: FT3: free triiodothyronine; FT4: free thyroxine; TSH: thyroid-stimulating hormone; FT3/FT4 ratio: FT3 to FT4 ratio; TFQI_FT3_: the thyroid feedback quantile-based index calculated by FT3; TFQI_FT4_: the thyroid feedback quantile-based index calculated by FT4; TPOAb: thyroid peroxidase antibody; TgAb: thyroglobulin antibody; eGFR: estimated glomerular filtration rate; TG: triglyceride; TC: total cholesterol; HDL-C: high-density lipoprotein-cholesterol; LDL-C: low-density lipoprotein-cholesterol.

The SPEED-Shunde study included 8319 participants with a mean age of 46 (SD 12, max 88, min 18). Among these participants, 12.30% were found to have diabetes, 38.19% were men, and 85.88% were non-smokers, with a mean FPG, HbA1c, PPG, FT3 and FT4 of 4.79 mmol/L, 5.68%, 7.77 mmol/L, 5.35 pmol/L and 11.26 pmol/L, respectively, and a medium TSH of 1.74 mIU/L.

### Associations of thyroid sensitivity indices with the prevalence of diabetes

Table 2 shows associations between quantiles of thyroid sensitivity indices and diabetes. In the NHANES, compared with participants in quartile 1st (Q1), those in quartile 4th (Q4) of TFQI_FT3_ (OR 2.12, 95% CI (1.18, 3.81)) (*p* < 0.05) and those in quartile 3rd (Q3) of TFQI_FT4_ (OR 2.31, 95% CI (1.18, 4.53)) (*p* < 0.05) were respectively associated with a greater prevalence of diabetes after adjusting for sex, age, BMI, eGFR, race/ethnicity, hypertension, thyroiditis, dyslipidemia, abused drink, education, smoking status and HOMA-IR. No associations between the FT3/FT4 ratio and the prevalence of diabetes were found.

**Table 2.** Logistic regression analysis for the association between thyroid sensitivity indices and diabetes in the euthyroid population.

	NHANES			SPEED-Shunde		
	Range	OR (95%CI)	*p*	Range	OR (95%CI)	*p*
**FT3/FT4 ratio**						
*P* for trend			.975			.252
Q4	0.55≤ Q4	0.89 (0.53, 1.50)	.650	0.53≤ Q4	1.07 (0.87, 1.30)	.535
Q3	0.49 ≤ Q3 < 0.55	1.31 (0.72, 2.38)	.356	0.48 ≤ Q3 < 0.53	1.16 (0.95, 1.41)	.148
Q2	0.44≤ Q2 < 0.49	1.05 (0.60, 1.83)	.854	0.43≤ Q2 < 0.48	0.96 (0.78, 1.18)	.710
Q1	Q1 < 0.44	Ref.		Q1 < 0.43	Ref.	
**TFQI_FT3_**						
*P* for trend			.011			.001
Q4	0.24≤ Q4	2.12 (1.18, 3.81)	.014	0.23≤ Q4	1.36 (1.11, 1.66)	.003
Q3	−0.02 ≤ Q3 < 0.24	2.22 (1.29, 3.83)	.006	−0.04 ≤ Q3 < 0.23	1.06 (0.87, 1.30)	.572
Q2	−0.32≤ Q2 < −0.02	1.92 (0.98, 3.74)	.056	−0.34≤ Q2 < −0.04	0.96 (0.78, 1.17)	.660
Q1	Q1 < −0.32	Ref.		Q1 < −0.34	Ref.	
**TFQI_FT4_**						
*P* for trend			.021			.137
Q4	0.19≤ Q4	1.79 (0.95, 3.37)	.071	0.20≤ Q4	1.19 (0.98, 1.46)	.083
Q3	−0.06 ≤ Q3 < 0.19	2.31 (1.18, 4.53)	.017	−0.04 ≤ Q3 < 0.20	1.15 (0.95, 1.40)	.161
Q2	−0.34≤ Q2 < −0.06	1.49 (0.65, 3.41)	.339	−0.32≤ Q2 < −0.04	1.22 (1.00, 1.48)	.046
Q1	Q1 < −0.34	Ref.		Q1 < −0.32	Ref.	

*Notes:* The model was adjusted for sex, age, BMI, eGFR, hypertension, thyroiditis, dyslipidemia, abused drink, education, smoking status and HOMA-IR in the SPEED-Shunde (further adjusted for race/ethnicity in the NHANES).

In the SPEED-Shunde, compared with participants in Q1, those in Q4 of TFQI_FT3_ had a greater prevalence of diabetes (OR 1.36, 95% CI (1.11, 1.66) (*p* < 0.05) after adjusting for sex, age, BMI, eGFR, hypertension, thyroiditis, dyslipidemia, abused drink, education, smoking status and HOMA-IR. However, no significant associations of TFQI_FT4_ and FT3/FT4 ratio with the prevalence of diabetes were found.

### Non-linear relationships of thyroid sensitivity indices with diabetes

**Figure 2.**
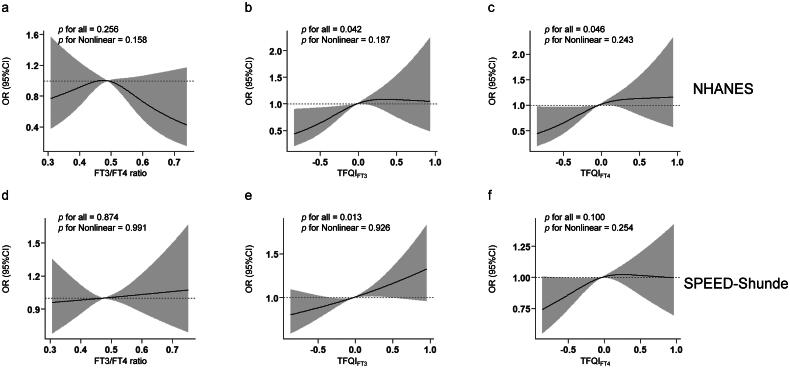
Non-linear relationships of thyroid sensitivity indices with diabetes. Restricted cubic spline (RCS) analysis was conducted. (a) FT3/FT4 ratio and diabetes in the NHANES (b) TFQI_FT3_ and diabetes in the NHANES (c) TFQI_FT4_ and diabetes in the NHANES (d) FT3/FT4 ratio and diabetes in the SPEED-Shunde (e) TFQI_FT3_ and diabetes in the SPEED-Shunde (f) TFQI_FT4_ and diabetes in the SPEED-Shunde. The model was adjusted for sex, age, BMI, eGFR, hypertension, thyroiditis, dyslipidemia, abused drink, education, smoking status and HOMA-IR in the SPEED-Shunde (further adjusted for race/ethnicity in the NHANES).

In Figure 2, we conducted an RCS analysis to examine the non-linear relationships between thyroid sensitivity indices and diabetes in both populations. We found that TFQI_FT3_ were linearly and positively associated with diabetes after adjusting for sex, age, BMI, eGFR, education, smoking status, abused drink, thyroiditis, hypertension, dyslipidemia and HOMA-IR (and further for race/ethnicity in the NHANES) in two populations (*P* for overall <0.05, *P* for non-linearity >0.05) **(**Figure 2(b,e)). Additionally, in the NHANES but not in the SPEED-Shunde, a linear relationship between TFQI_FT4_ and diabetes was found (Figure 2(c,f)). No associations between FT3/FT4 ratio and diabetes were found in either population (Figure 2(a,d)).

### Associations of thyroid sensitivity indices with diabetes stages

**Figure 3.**
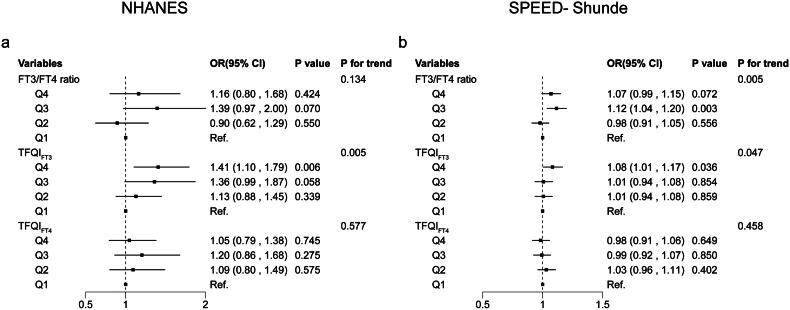
Associations of thyroid sensitivity indices with diabetes stages using general ordinal logit regression. Thyroid sensitivity indices quartiles and diabetes (a) in the NHANES and (b) in the SPEED-Shunde. The model was adjusted for sex, age, BMI, eGFR, hypertension, thyroiditis, dyslipidemia, abused drink, education, smoking status and HOMA-IR in the SPEED-Shunde (further adjusted for race/ethnicity in the NHANES).

In the general ordinal logit regression model, compared with the first quartile of TFQI_FT3_, the fourth quartile of TFQI_FT3_ was associated with a 41% (OR 1.41, 95% CI (1.10, 1.79)) (*p* < 0.05) higher odds of a higher glucose status (diabetes vs. prediabetes and prediabetes vs. normoglycemia) in the NHANES and an 8% higher odds of a higher glucose category (OR 1.08, 95% CI (1.01, 1.17)) (*p* < 0.05) in the SPEED-Shunde (Figure 3). The model was adjusted with sex, age, BMI, eGFR, hypertension, thyroiditis, dyslipidemia, abused drink, education, smoking status and HOMA-IR in the SPEED-Shunde and further adjusted for race/ethnicity in the NHANES. However, no associations of TFQI_FT4_ and FT3/FT4 ratio with the odds of the higher glucose status (diabetes vs. prediabetes and prediabetes vs. normoglycemia) were found (both *p* > 0.05).

### Stratification analysis

**Figure 4.**
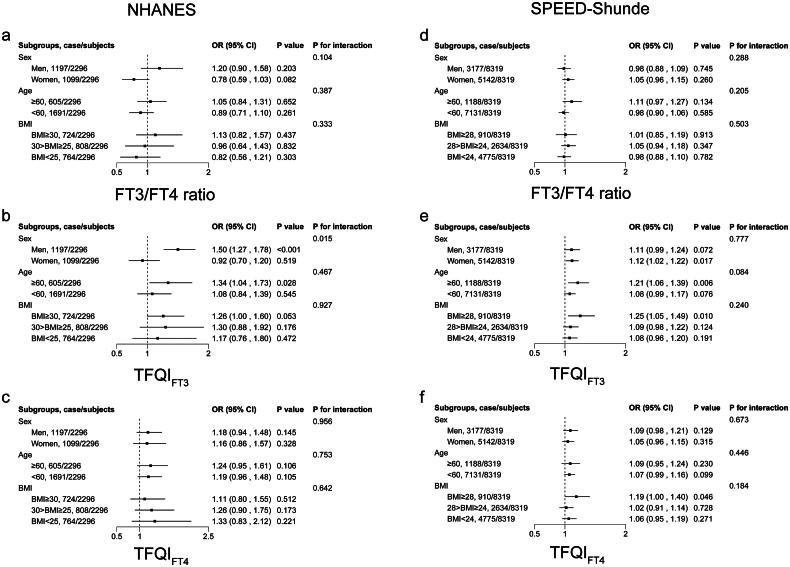
Stratification analysis stratified by sex, age and BMI. Associations of (a) FT3/FT4 ratio, (b) TFQI_FT3_ and (c) TFQI_FT4_ with diabetes in the NHANES. Associations of (d) FT3/FT4 ratio, (e) TFQI_FT3_ and (f) TFQI_FT4_ with diabetes in the SPEED-Shunde. The model was adjusted for sex, age, BMI, eGFR, hypertension, thyroiditis, dyslipidemia, abused drink, education, smoking status and HOMA-IR in the SPEED-Shunde (further adjusted for races in the NHANES).

Figure 4 shows the results of the stratification analysis. In the NHANES, participants with higher TFQI_FT3_ had a higher prevalence of diabetes, especially among men (OR 1.50, 95% CI (1.27, 1.78)) (*p* < 0.05), those with age ≥ 60 years (OR 1.34, 95% CI (1.04, 1.73)) (*p* < 0.05) and those BMI ≥30 kg/m^2^ (OR 1.26, 95% CI (0.997, 1.60)) (*p* = 0.053). The associations remained consistent in the SPEED-Shunde.

## Discussion

To the best of our knowledge, this is the first study to evaluate the association of TFQI_FT3_ with the prevalence of diabetes in a large sample of euthyroid adults in the United States and China. We found the consistently positive associations of TFQI_FT3_ levels with the prevalence of diabetes and the proportional odds of the higher glucose status both in the euthyroid adults in the United States and China, particularly in the elderly or obese participants, while TFQI_FT4_ was positively associated with the prevalence of diabetes in the euthyroid adults in the United States, but not in China. Our findings implied the importance of TFQI_FT3_, an indicator of impaired central sensitivity to FT3, for predicting diabetes.

Prior studies have presented contradictory findings regarding the relationship between diabetes and the single hormones of FT3, FT4 and TSH [4,20]. For example, one early study suggested that decreased FT3 and increased FT4 levels are independently related to a higher prevalence of diabetes in both males and females, and TSH is inversely related to T2DM in males only [20,37]. However, one recent study found that high TSH and low FT4 levels in the reference range were significantly associated with the prevalence of diabetes [4]. These contradictory findings can be attributed to the interconnectedness of these hormones. As a result, the investigation of composite indices, such as TFQI_FT3_ or TFQI_FT4_, has been deemed necessary to offer a comprehensive understanding of the regulation of thyroid hormone homeostasis, surpassing the limitations of relying solely on a single hormone [10,38].

Laclaustra et al. [12] proposed a positive association between higher TFQI_FT4_ levels and the prevalence of diabetes in a cross-sectional study conducted in the United States. However, a recent study involving 3573 Chinese participants did not observe a significant association between TFQI_FT4_ and diabetes [39]. These findings were similar to our results that TFQI_FT4_ was positively associated with the prevalence of diabetes in euthyroid adults in the United States rather than in China. In addition, previous studies have reported significant associations of TFQI_FT3_, but not TFQI_FT4_, with MAFLD and NAFLD [19,40–42]. However, investigations into the associations between TFQI_FT3_ and diabetes were limited. This study presents novel findings indicating a positive association between TFQI_FT3_ levels and the prevalence of diabetes in euthyroid adults from both the United States and China. These consistent associations indicated that TFQI_FT3_ may have a more reliable predictive ability for diabetes than TFQI_FT4_.

Notably, our study revealed that the associations between TFQI_FT3_ and diabetes prevalence remained significant only among participants aged over 60 or obese in the United States and China. This observation may be attributed to the higher likelihood of elevated FT3 and TSH levels in elderly and obese individuals. A study demonstrated a negative association between age and levels of TSH and FT3, indicating a potentially significant alteration in the feedback regulation of thyroid function in older people [43]. Furthermore, another study revealed that a genetically predisposed higher BMI is associated with elevated FT3 levels, while no significant correlation was observed with FT4 levels [44]. Collectively, these studies suggest that TFQI_FT3_ may hold greater importance than TFQI_FT4_ in elderly and obese populations.

Possible explanations for the relatively consistent associations between TFQI_FT3_ and diabetes primarily encompass the compensatory rise in serum FT3 levels and the physiological impacts of FT3 and TSH. Firstly, the heightened levels of serum FT3, predominantly derived from serum FT4 through deiodinase conversion, can be perceived as a compensatory and adaptive mechanism to augment energy expenditure in metabolic disorders, such as obesity [45]. This hypothesis was supported by a previous study where FT3 levels decreased after weight loss [46]. Furthermore, a previous study demonstrated a significant elevation in TSH levels among mice subjected to a high-fat diet, potentially attributed to a reduction in the central sensitivity to thyroid hormones [47]. Secondly, it has been suggested that FT3 may contribute to augmented hepatic glucose production and intestinal glucose absorption, diminished muscle glycogen storage, upregulated glycogenolysis and intensified lipolysis, ultimately resulting in heightened insulin resistance [48]. Thirdly, it has been observed that TSH could induce leptin secretion in human adipose tissue, which reduced insulin secretion and synthesis in pancreatic β-cells [49]. Consequently, the composite index TFQI_FT3_, which incorporates both TSH and FT3, may provide a more accurate indication of the likelihood of developing diabetes compared to the individual index in individuals with normal TSH, FT3 and FT4 levels.

The present study possesses notable strengths, primarily from its relatively substantial sample size and the evidence obtained from two distinct cohorts recruited from the United States and China. Nonetheless, certain limitations persist. Firstly, despite the utilization of electrochemiluminescence immunoassays in quality-driven laboratory settings in both cohorts, it is imperative to acknowledge the possibility of an inherent influence on the outcomes due to variations in laboratory assays for the quantification of TSH, FT3, FT4 and glucose. Secondly, despite our efforts to control for various confounding factors, it is important to acknowledge the possibility of residual and recall bias influencing the effect estimation. Thirdly, the study’s cross-sectional design limits its ability to establish causal relationships between thyroid hormone sensitivity and diabetes. Therefore, it is essential to consider the reverse link from diabetes and glucose metabolism to thyroid hormone resistance and to conduct further prospective cohort investigations or Mendelian randomization research to explore the potential causal inferences.

In summary, our study revealed a significantly and consistently positive association between TFQI_FT3_ and diabetes in euthyroid adults from the United States and China, particularly in elderly and obese populations. Conversely, we observed a positive association between TFQI_FT4_ and diabetes solely in euthyroid adults from the United States, with no such association found in China. These findings provide evidence that the association between TFQI_FT3_ and diabetes may have broader applicability to different race/ethnicity, which warrants further prospective cohort studies.

## Data Availability

The data supporting the study findings are available from the corresponding authors upon reasonable request.
